# Evaluation of Direct Examination, Culture, and Histopathology in the Diagnosis of Mucormycosis: Reiterating the Role of KOH Mount for Early Diagnosis

**DOI:** 10.7759/cureus.19455

**Published:** 2021-11-10

**Authors:** Aroop Mohanty, Pratima Gupta, Kunnumbrath Arathi, Shalinee Rao, Ranjana Rohilla, Suneeta Meena, Ashok Singh, Neelam Kaistha, Rama S Rath, Saurabh Varshney

**Affiliations:** 1 Clinical Microbiology, All India Institute of Medical Sciences (AIIMS) Gorakhpur, Gorakhpur, IND; 2 Microbiology, All India Institute of Medical Sciences (AIIMS) Rishikesh, Rishikesh, IND; 3 Pathology, Employees' State Insurance Corporation (ESIC) Hospital Chennai, Chennai, IND; 4 Pathology, All India Institute of Medical Sciences (AIIMS) Rishikesh, Rishikesh, IND; 5 Microbiology, Shri Guru Ram Rai Institute of Medical and Health Sciences, Dehradun, IND; 6 Laboratory Medicine, All India Institute of Medical Sciences (AIIMS) Delhi, Delhi, IND; 7 Community Medicine, All India Institute of Medical Sciences (AIIMS) Gorakhpur, Gorakhpur, IND; 8 Otolaryngology, All India Institute of Medical Sciences (AIIMS) Deoghar, Deoghar, IND

**Keywords:** invasive fungal infection, histopathology examination, fungal culture, koh preparation, “mucormycosis”

## Abstract

Introduction

Invasive fungal infections have always been a major cause of mortality and morbidity and are especially prevalent in the immunosuppressed groups of patients. Members of the *Mucoracea *family have an increasing incidence and prevalence. It has always been difficult to diagnose this condition due to various reasons.

Materials and Methods

This was an observational study carried out jointly by the Department of Microbiology and the Department of Pathology for a duration of one year. All patients who presented in various clinical departments with a high index of clinical suspicion for mucormycosis were included in this study. A total of 186 samples were received from suspected cases of mucormycosis and were all subjected to direct microscopy by potassium hydroxide (KOH), fungal culture, and histopathological examination.

Results

Mucormycosis was documented in 33 out of 186 cases on direct microscopy, whereas 21 were positive on fungal culture. Histopathological positivity was reduced with only 11 cases showing aseptate hyphae suggestive of mucormycosis.

Conclusion

As these organisms generally do not grow well on routine culture media and with the histopathological results also being not suggestive clearly of mucormycosis, direct microscopy thus becomes more important and essential in the rapid diagnosis of this deadly condition.

## Introduction

Mucormycosis is a reemerging fungal infection and has been seen increasingly in immunocompromised patients. It is caused by fungi belonging to the order Mucorales [1]. Although mucormycosis has been associated with high morbidity and mortality, its prevalence has been enormous. This disease has been in limelight since the outbreak of COVID-19 in November 2020. The immune dysregulation caused by the virus and the concurrent use of immunomodulatory drugs has increased the risk of these fungal infections in such patients [2]. Among all the fungal infections, COVID-19-associated mucormycosis (CAM) has led to most deaths and caused a lot of suffering worldwide. As India has the second largest population with diabetes mellitus in the world, mucormycosis has seen a steep rise mostly in patients with uncontrolled diabetes mellitus with an estimated prevalence being 140 cases per million population.

It has always been a challenge to diagnose this condition, and the main prerequisites for an accurate diagnosis are a high index of suspicion and recognition of host factors along with a prompt assessment of clinical manifestations [3,4]. Microscopy along with culture and histopathology demonstration of Mucorales have been the mainstay of diagnosis, albeit with less sensitivity. It is based upon the isolation and identification of the fungus responsible for the infection. Various specimens can be considered depending upon the clinical manifestations and the site of infection, but tissue biopsy remains the sample of choice and always gives the best results. Direct microscopy of a fresh tissue sample with a few drops of potassium hydroxide (KOH) helps us in identifying the typical pauciseptate/aseptate ribbon-like hyphae and gives us a presumptive diagnosis. It is of utmost importance as early treatment with specific antifungal drugs can be initiated to prevent a fatal outcome. Furthermore, the use of fluorochromes (Calcofluor White or Blankophor P) and silver stains (Gomori-Grocott) can be used to increase the sensitivity in cases of low fungal density [5]. In addition to direct microscopy, histopathological diagnosis is a very important diagnostic tool as it distinguishes a fungal pathogen from a culture contaminant. However, histopathological result conveys only morphological diagnosis and lacks information about the species of the causative organism. Complete speciation can be done with the help of fungal culture, which is the gold standard for this disease. The combination of histopathology features, direct microscopy, and culture results assists in correctly identifying Mucorales causing mucormycosis. This study was conducted with the objective of evaluating the role of microscopy along with culture and histopathology in providing an accurate diagnosis of mucormycosis.

## Materials and methods

This study was carried out for a duration of one year (January 2019-December 2019) in a tertiary care hospital situated in Uttarakhand to determine the prevalence of mucormycosis in the region. A retrospective data analysis was done from the medical records of the patients. All patients who presented in various clinical departments with a high index of clinical suspicion for mucormycosis were included in this study. The enrolled patients comprised of both immunocompromised and immunocompetent individuals. The specimens from the patients were collected in two sterile containers, one with and the other without 10% formalin, and processed in the Department of Microbiology and the Department of Pathology, respectively. The testing of the samples in the Department of Microbiology included direct potassium hydroxide (KOH) examination and fungal culture. The samples were inoculated without crushing in two tubes containing Sabouraud's dextrose agar (SDA) without antibiotics, with one tube incubated at 37°C and the other at 25°C. The cultures were examined for growth daily for the first week and twice a week for the subsequent period. The fungal isolates were finally identified by conventional techniques such as lactophenol cotton blue (LCB) mount. For histopathological examination, tissue sections were stained with hematoxylin and eosin (H&E), periodic acid-Schiff (PAS) stain, and Grocott methenamine silver (GMS) stain.

The diagnosis of mucormycosis was confirmed when broad aseptate/sparsely septate, ribbon-like hyphae with right (wide) angle branching were demonstrated in the specimen with or without isolation of mucormycetes. The diagnosis was also confirmed by suggestive histopathological findings.

Analysis

Data were entered in Microsoft Excel 2021, and analysis was done using the STATA-12. Agreement between the tests was calculated using Kappa. The agreement was classified using standard classification methods. Sensitivity and specificity were calculated using the standard formula with 95% confidence intervals.

## Results

In the one-year (2019) study period carried out jointly by the Department of Microbiology and the Department of Pathology, a total of 186 clinical samples with a high index of clinical suspicion for mucormycosis were collected. Of these, 60% (111/186) were males and 40% (75/186) were females. The mean age of patients was 40 years. The majority of the samples received were nasal (122), followed by ear (43), mucosa from the sphenoid cavity (5), pus from neck node (3), sputum (2), hard palate biopsy (2), tissue from functional endoscopic sinus surgery (FESS) and cortical mastoidectomy (2), glossectomy (2), submandibular gland (1), post-aural granulation tissue (1), pericardial tissue (1), pus swab from left aryepiglottic growth (1), and biopsy from the left side of the face (1). On direct microscopy by KOH mount, 33 out of 186 (17.2%) turned out to be positive, while 21 out of 186 (11.3%) showed growth on fungal culture. On the other hand, histopathology was positive only in 11 out of the total 186 (6%) cases.

Table 1 shows the distribution of cases diagnosed by different techniques. The maximum cases were diagnosed by positive direct KOH examination alone, i.e. 6.45% (12/186), least by positive histopathology alone, i.e., only 0.53% (01/186).

**Table 1:** Distribution of cases diagnosed by different techniques

S. no.	Diagnosed by			(n = 186)
1	Culture isolation	KOH microscopy	Histopathology	Detection rate (no. positive)
2	+	+	+	2.15% (04)
3	+	+	-	5.91% (11)
4	+	-	-	3.22% (06)
5	+	-	+	0% (0)
6	-	+	-	6.45% (12)
7	-	+	+	3.22% (06)
8	-	-	+	0.53% (01)

As per Table 2, there is a moderate agreement between culture and KOH microscopy (kappa = 0.5). Table 3 shows the test characteristics of the KOH examination and histopathology. However, the sensitivity and specificity of the KOH microscopy are 64% and 91%, respectively. The agreement between culture and histopathology is 0.2, and the sensitivity and specificity of histopathology are 20% and 96%, respectively. In addition, KOH microscopy had a good negative predictive value of 94.23%.

**Table 2:** Level of agreement between culture, KOH result, and histopathology

Diagnostic modalities	Kappa	P value
Culture versus KOH	0.4970	0.000
Culture versus histopathology	0.2135	0.001

**Table 3:** Test characteristics of KOH and histopathology

Test	KOH microscopy	Histopathology
Sensitivity	64% (42.52–82.03)	20% (6.83–40.70)
Specificity	90.74% (85.19–94.72)	96.30% (92.11–98.63)
Positive predictive value	51.61% (37.75–65.23)	45.45% (21.55–71.65)
Negative predictive value	94.23% (90.62–96.50)	88.64% (86.48–90.49)

## Discussion

During the last 10 years, there has been an enormous surge in the occurrence of mucormycosis cases worldwide, largely as a result of the expansion of the population at risk of having predisposing conditions. In our study, 60% were males and 40% were females, with a mean age of 40 years. Young age males are more prone to acute necrotizing infection. This was in concordance with a study conducted by Bala et al., where the incidence was commoner in males (72%) than in females (28%), and the mean age of the patients was 40.43 years [6]. In another study from a tertiary care center in North India, wherein 388 proven/probable cases of mucormycosis were included, the male/female ratio was reported to be 2.3:1, and the median age of the patients was 45.5 years [7].

Rhinocerebral mucormycosis has been found to be the commonest clinical presentation in most of the published literature, and the same was noted in the present study. Similar findings have been observed in a prospective multicenter study and also in another study conducted in the western part of India [7,8]. A review by Prakash et al. also states rhino-orbital presentation as the commonest presentation and is associated with significant morbidity and mortality [9].

On careful analysis of the results, several reasons could be postulated for the low rate of positivity in histopathology as compared with microbiological diagnosis by direct microscopy and culture. One of the reasons could be the sample sent to the Department of Microbiology laboratory and to the histopathology not being from the exact same site and therefore may not be equally representative. It is very challenging to diagnose fungal infections on scanty tissue sections; it requires good expertise [10]. Another reason could be missing the organism, resulting in underdiagnosis. In large specimens, grossing and biting may not be representative, or organisms may be present in the deeper plane and could have been identified on viewing deeper sections. Tissue reactions can be considered a soft sign to suspect fungal infections such as necrosis, histiocytes and lymphocytes, eosinophil aggregates, giant cells, and sometimes neutrophil clumps [11]. Tissue response is variable and based on allergic, acute, or chronic infection [10]. Mucormycosis tends to be tissue and vascular invasive [12]. This study highlights the fact that communication between microbiologists, clinicians, and pathologists is essential to reduce the incidence of underdiagnosis. The value of histopathology has been emphasized in implying the real significance of fungal colonies in direct microscopy and culture as to whether it is pathogenic or contaminant, as well as invasive or noninvasive, in nature [10].

KOH along with histopathology is recommended by the European Confederation of Medical Mycology in cooperation with the Mycoses Study Group Education and Research Consortium (ECMM/MSG ERC) [13]. KOH microscopy, if done intraoperatively, can help delineate clear surgical margins. A rapid diagnosis available through KOH may help clinicians initiate antifungal treatment early in the disease course for a better and favorable outcome. Although it involves sampling through an invasive method, unfortunately, there is no alternative to it at present [14]. Some studies have also used the semi-nested PCR method in fresh tissue samples and have obtained reliable results.

## Conclusions

KOH mount microscopy is a reasonably priced and irreplaceable method to make a rapid presumptive diagnosis. It has been for ages the best means to diagnose this fatal condition and provide a presumptive guide to the clinicians in their management. It helps them in initiating the appropriate treatment and therefore saving precious time. Being a point of care test that can be performed immediately, it is the fastest means of getting a presumptive diagnosis. Nevertheless, new molecular and antigenic tools are the need of the hour for the early detection of mucormycosis and therapeutic monitoring.
